# {*N*-Methyl-*N*′-[1-(pyridin-2-yl)ethyl­idene]ethane-1,2-diamine-κ^3^ *N*,*N*′,*N*′′}­bis(thio­cyanato-κ*N*)zinc(II)

**DOI:** 10.1107/S1600536811019945

**Published:** 2011-06-04

**Authors:** Xian-Wen Li

**Affiliations:** a Department of Chemistry and Chemical Engineering, Minjiang University, Fuzhou 350108, People’s Republic of China

## Abstract

In the title compound, [Zn(NCS)_2_(C_10_H_15_N_3_)], the Zn atom is five-coordinated by the three N-donor atoms of the Schiff base ligand and by two N atoms from two thio­cyanate anions, forming a distorted ZnN_5_ trigonal–bipyramidal coordination geometry for the metal ion. The side chain of the ligand is disordered over two sets of sites in a 0.655 (12):0.345 (12) ratio. In the crystal, mol­ecules are linked by N—H⋯S hydrogen bonds, generating [100] chains.

## Related literature

For the biological activity of Schiff base compounds, see: Panneerselvam *et al.* (2005 ▶); Shi *et al.* (2007 ▶); Singh *et al.* (2006 ▶, 2007 ▶); Zhong *et al.* (2006 ▶). For the Schiff base complexes we reported previously, see: Li & Qiu (2008*a* ▶,*b* ▶).
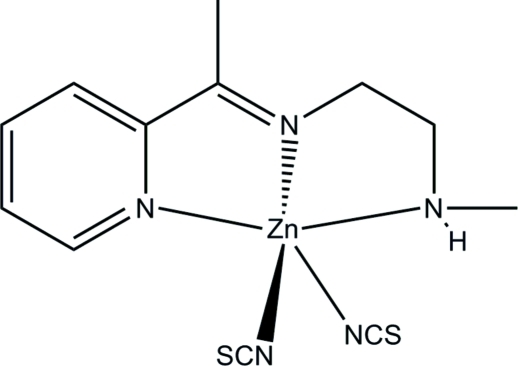


## Experimental

### 

#### Crystal data

[Zn(NCS)_2_(C_10_H_15_N_3_)]
*M* *_r_* = 358.78
Monoclinic, 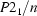

*a* = 7.6674 (3) Å
*b* = 14.8062 (5) Å
*c* = 14.3766 (6) Å
β = 101.853 (2)°
*V* = 1597.30 (11) Å^3^
*Z* = 4
Mo *K*α radiation
μ = 1.80 mm^−1^
*T* = 298 K
0.20 × 0.20 × 0.18 mm

#### Data collection

Bruker APEXII CCD diffractometer
Absorption correction: multi-scan (*SADABS*; Sheldrick, 2004 ▶) *T*_min_ = 0.715, *T*_max_ = 0.738
9004 measured reflections
3352 independent reflections
2095 reflections with *I* > 2σ(*I*)
*R*_int_ = 0.029

#### Refinement

*R*[*F* ^2^ > 2σ(*F* ^2^)] = 0.040
*wR*(*F* ^2^) = 0.105
*S* = 1.02
3352 reflections
201 parameters
12 restraints
H-atom parameters constrained
Δρ_max_ = 0.46 e Å^−3^
Δρ_min_ = −0.46 e Å^−3^

### 

Data collection: *APEX2* (Bruker, 2004 ▶); cell refinement: *SAINT* (Bruker, 2004 ▶); data reduction: *SAINT*; program(s) used to solve structure: *SHELXS97* (Sheldrick, 2008 ▶); program(s) used to refine structure: *SHELXL97* (Sheldrick, 2008 ▶); molecular graphics: *SHELXTL* (Sheldrick, 2008 ▶); software used to prepare material for publication: *SHELXTL*.

## Supplementary Material

Crystal structure: contains datablock(s) global, I. DOI: 10.1107/S1600536811019945/hb5892sup1.cif

Structure factors: contains datablock(s) I. DOI: 10.1107/S1600536811019945/hb5892Isup2.hkl

Additional supplementary materials:  crystallographic information; 3D view; checkCIF report

## Figures and Tables

**Table 1:** Selected bond lengths (Å)

Zn1—N5	1.974 (4)
Zn1—N4	1.986 (4)
Zn1—N2	2.088 (3)
Zn1—N3	2.163 (4)
Zn1—N1	2.195 (3)

**Table 2:** Hydrogen-bond geometry (Å, °)

*D*—H⋯*A*	*D*—H	H⋯*A*	*D*⋯*A*	*D*—H⋯*A*
N3—H3*A*⋯S1^i^	0.91	2.66	3.551 (5)	165

Symmetry code: (i) 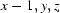.

**Fig. 1.**
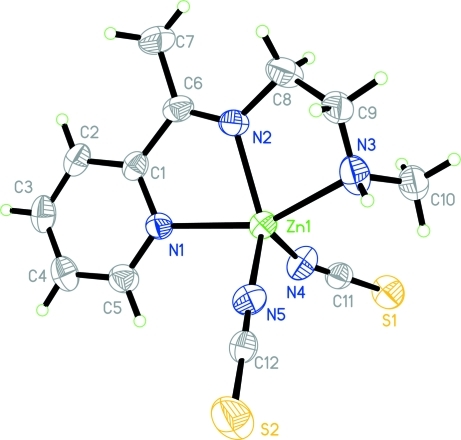
The molecular structure of the title compound, showing 30% probability displacement ellipsoids. Only the major component of the disordered group is shown.

## supplementary crystallographic information

### Comment

Schiff base compounds have been reported to have excellent biological activity (Shi *et al.*, 2007; Panneerselvam *et al.*, 2005). The metal complexes derived from the Schiff bases also have excellent biological activity (Singh *et al.*, 2006, 2007; Zhong *et al.*, 2006). As a continuation of our work on Schiff base complexes (Li & Qiu, 2008*a*,*b*), we report herein the crystal structure of the title zinc complex, (I).

In the title mononuclear zinc(II) complex, the Zn atom is five-coordinated by the three donor atoms (N1, N2, and N3) of the Schiff baes ligand, and two N atoms from two thiocyanate ligands, forming a slightly distorted trigonal-bipyramidal geometry (Fig. 1). The coordinate bond values (Table 1) are within normal ranges.

### Experimental

The title compound was obtained by the reaction of equimolar quantities (0.1 mmol each) of 2-acetylpyridine, *N*-methylethane-1,2-diamine, sodium thiocyanate, and zinc acetate in ethanol. Colorless blocks of (I) were obtained by the slow evaporation of the filtrate in air.

### Refinement

H atoms were positioned geometrically and refined using a riding model, with C—H = 0.93–0.97Å and with *U*_iso_(H) = 1.2 (1.5 for methyl groups) times *U*_eq_(C).

The amino H atoms were located in a difference map and refined with N—H distance restrained to 0.90 (1) Å. The remaining H atoms were positioned geometrically (C—H = 0.93–0.97 Å, N—H = 0.91 Å) and refined using a riding model, with *U*_iso_(H) = 1.2*U*_eq_(C) and 1.5*U*_eq_(methyl C). The C9—N3—C10 moiety is disordered over two sites, with occupancies of 0.655 (3) and 0.345 (3).

### Crystal data

**Table:**

[Zn(NCS)_2_(C_10_H_15_N_3_)]	*F*(000) = 736
*M**_r_* = 358.78	*D*_x_ = 1.492 Mg m^−^^3^
Monoclinic, *P*2_1_/*n*	Mo *K*α radiation, λ = 0.71073 Å
Hall symbol: -P 2yn	Cell parameters from 2372 reflections
*a* = 7.6674 (3) Å	θ = 2.6–25.1°
*b* = 14.8062 (5) Å	µ = 1.80 mm^−^^1^
*c* = 14.3766 (6) Å	*T* = 298 K
β = 101.853 (2)°	Block, colorless
*V* = 1597.30 (11) Å^3^	0.20 × 0.20 × 0.18 mm
*Z* = 4	

### Data collection

**Table:**

Bruker APEXII CCD diffractometer	3352 independent reflections
Radiation source: fine-focus sealed tube	2095 reflections with *I* > 2σ(*I*)
graphite	*R*_int_ = 0.029
ω scans	θ_max_ = 26.7°, θ_min_ = 2.0°
Absorption correction: multi-scan (*SADABS*; Sheldrick, 2004)	*h* = −9→9
*T*_min_ = 0.715, *T*_max_ = 0.738	*k* = −17→18
9004 measured reflections	*l* = −18→11

### Refinement

**Table:**

Refinement on *F*^2^	Primary atom site location: structure-invariant direct methods
Least-squares matrix: full	Secondary atom site location: difference Fourier map
*R*[*F*^2^ > 2σ(*F*^2^)] = 0.040	Hydrogen site location: inferred from neighbouring sites
*wR*(*F*^2^) = 0.105	H-atom parameters constrained
*S* = 1.02	*w* = 1/[σ^2^(*F*_o_^2^) + (0.0323*P*)^2^ + 1.952*P*] where *P* = (*F*_o_^2^ + 2*F*_c_^2^)/3
3352 reflections	(Δ/σ)_max_ = 0.001
201 parameters	Δρ_max_ = 0.46 e Å^−^^3^
12 restraints	Δρ_min_ = −0.46 e Å^−^^3^

### Special details

**Table:**

Geometry. All e.s.d.'s (except the e.s.d. in the dihedral angle between two l.s. planes) are estimated using the full covariance matrix. The cell e.s.d.'s are taken into account individually in the estimation of e.s.d.'s in distances, angles and torsion angles; correlations between e.s.d.'s in cell parameters are only used when they are defined by crystal symmetry. An approximate (isotropic) treatment of cell e.s.d.'s is used for estimating e.s.d.'s involving l.s. planes.
Refinement. Refinement of *F*^2^ against ALL reflections. The weighted *R*-factor *wR* and goodness of fit *S* are based on *F*^2^, conventional *R*-factors *R* are based on *F*, with *F* set to zero for negative *F*^2^. The threshold expression of *F*^2^ > σ(*F*^2^) is used only for calculating *R*-factors(gt) *etc*. and is not relevant to the choice of reflections for refinement. *R*-factors based on *F*^2^ are statistically about twice as large as those based on *F*, and *R*- factors based on ALL data will be even larger.

### Fractional atomic coordinates and isotropic or equivalent isotropic displacement parameters (Å^2^)

**Table:**

	*x*	*y*	*z*	*U*_iso_*/*U*_eq_	Occ. (<1)
Zn1	0.72087 (6)	−0.07353 (3)	0.71636 (3)	0.05504 (17)	
S1	1.28221 (17)	−0.15205 (10)	0.89256 (10)	0.0835 (4)	
S2	0.3366 (2)	−0.32564 (11)	0.66375 (10)	0.1063 (6)	
N1	0.7424 (4)	−0.0555 (2)	0.5676 (2)	0.0524 (8)	
N2	0.6777 (4)	0.0641 (2)	0.6890 (2)	0.0559 (8)	
N4	0.9697 (5)	−0.1140 (3)	0.7651 (3)	0.0760 (11)	
N5	0.5583 (5)	−0.1782 (2)	0.6923 (2)	0.0684 (10)	
C1	0.7390 (5)	0.0309 (3)	0.5392 (3)	0.0515 (9)	
C2	0.7683 (5)	0.0539 (3)	0.4504 (3)	0.0628 (11)	
H2	0.7670	0.1142	0.4319	0.075*	
C3	0.7993 (6)	−0.0131 (4)	0.3901 (3)	0.0708 (13)	
H3	0.8193	0.0015	0.3303	0.085*	
C4	0.8009 (6)	−0.1007 (4)	0.4174 (3)	0.0706 (13)	
H4	0.8207	−0.1469	0.3770	0.085*	
C5	0.7719 (6)	−0.1192 (3)	0.5074 (3)	0.0629 (11)	
H5	0.7731	−0.1792	0.5268	0.075*	
C6	0.7014 (5)	0.0970 (3)	0.6107 (3)	0.0577 (10)	
C7	0.6971 (7)	0.1958 (3)	0.5876 (4)	0.0877 (15)	
H7A	0.6442	0.2284	0.6325	0.132*	
H7B	0.6279	0.2052	0.5247	0.132*	
H7C	0.8164	0.2172	0.5907	0.132*	
C11	1.1008 (6)	−0.1300 (3)	0.8180 (3)	0.0573 (10)	
C12	0.4653 (6)	−0.2395 (3)	0.6815 (3)	0.0586 (10)	
C8	0.6362 (7)	0.1164 (3)	0.7662 (3)	0.0810 (14)	0.655 (12)
H8A	0.5502	0.1631	0.7417	0.097*	0.655 (12)
H8B	0.7431	0.1451	0.8016	0.097*	0.655 (12)
C9	0.5577 (12)	0.0508 (5)	0.8318 (5)	0.074 (3)	0.655 (12)
H9A	0.5582	0.0803	0.8921	0.089*	0.655 (12)
H9B	0.4349	0.0371	0.8026	0.089*	0.655 (12)
C10	0.7978 (14)	−0.0366 (8)	0.9293 (6)	0.089 (3)	0.655 (12)
H10A	0.7586	−0.0160	0.9849	0.133*	0.655 (12)
H10B	0.8964	−0.0004	0.9196	0.133*	0.655 (12)
H10C	0.8346	−0.0986	0.9377	0.133*	0.655 (12)
N3	0.6540 (6)	−0.0290 (3)	0.8480 (2)	0.0814 (12)	0.655 (12)
H3A	0.5729	−0.0703	0.8591	0.098*	0.655 (12)
C8'	0.6362 (7)	0.1164 (3)	0.7662 (3)	0.0810 (14)	0.345 (12)
H8'A	0.5082	0.1233	0.7582	0.097*	0.345 (12)
H8'B	0.6893	0.1760	0.7673	0.097*	0.345 (12)
C9'	0.709 (2)	0.0678 (7)	0.8562 (7)	0.097 (7)	0.345 (12)
H9'A	0.6658	0.0959	0.9081	0.116*	0.345 (12)
H9'B	0.8382	0.0719	0.8702	0.116*	0.345 (12)
C10'	0.723 (2)	−0.0647 (13)	0.9393 (8)	0.066 (5)	0.345 (12)
H10D	0.6699	−0.0338	0.9854	0.099*	0.345 (12)
H10E	0.8497	−0.0565	0.9546	0.099*	0.345 (12)
H10F	0.6955	−0.1279	0.9400	0.099*	0.345 (12)
N3'	0.6540 (6)	−0.0290 (3)	0.8480 (2)	0.0814 (12)	0.345 (12)
H3'A	0.5331	−0.0302	0.8394	0.098*	0.345 (12)

### Atomic displacement parameters (Å^2^)

**Table:**

	*U*^11^	*U*^22^	*U*^33^	*U*^12^	*U*^13^	*U*^23^
Zn1	0.0644 (3)	0.0492 (3)	0.0518 (3)	0.0000 (2)	0.0128 (2)	0.0037 (2)
S1	0.0678 (8)	0.0948 (10)	0.0845 (9)	0.0061 (7)	0.0077 (6)	−0.0070 (7)
S2	0.1434 (14)	0.0975 (11)	0.0869 (9)	−0.0621 (10)	0.0447 (9)	−0.0190 (8)
N1	0.062 (2)	0.0456 (19)	0.0502 (18)	−0.0062 (15)	0.0126 (15)	−0.0006 (15)
N2	0.061 (2)	0.0461 (18)	0.061 (2)	0.0029 (16)	0.0128 (16)	−0.0033 (16)
N4	0.074 (3)	0.082 (3)	0.073 (3)	0.008 (2)	0.018 (2)	0.021 (2)
N5	0.082 (3)	0.055 (2)	0.069 (2)	−0.007 (2)	0.017 (2)	0.0074 (18)
C1	0.042 (2)	0.057 (2)	0.053 (2)	−0.0039 (18)	0.0023 (17)	0.0080 (19)
C2	0.055 (3)	0.071 (3)	0.059 (3)	−0.008 (2)	0.003 (2)	0.020 (2)
C3	0.064 (3)	0.100 (4)	0.048 (2)	−0.015 (3)	0.010 (2)	0.009 (3)
C4	0.068 (3)	0.092 (4)	0.054 (3)	−0.010 (3)	0.018 (2)	−0.012 (2)
C5	0.072 (3)	0.056 (3)	0.064 (3)	−0.005 (2)	0.022 (2)	−0.002 (2)
C6	0.054 (2)	0.044 (2)	0.070 (3)	−0.0030 (18)	0.002 (2)	0.004 (2)
C7	0.114 (4)	0.048 (3)	0.099 (4)	−0.001 (3)	0.015 (3)	0.008 (3)
C11	0.065 (3)	0.051 (2)	0.060 (3)	−0.005 (2)	0.024 (2)	0.003 (2)
C12	0.075 (3)	0.059 (3)	0.045 (2)	−0.002 (2)	0.022 (2)	0.007 (2)
C8	0.097 (4)	0.060 (3)	0.089 (4)	−0.004 (3)	0.025 (3)	−0.019 (3)
C9	0.081 (6)	0.080 (6)	0.062 (4)	0.033 (4)	0.018 (4)	−0.005 (4)
C10	0.111 (7)	0.092 (7)	0.059 (5)	0.030 (5)	0.010 (5)	−0.003 (4)
N3	0.092 (3)	0.097 (3)	0.057 (2)	−0.012 (3)	0.020 (2)	−0.009 (2)
C8'	0.097 (4)	0.060 (3)	0.089 (4)	−0.004 (3)	0.025 (3)	−0.019 (3)
C9'	0.096 (14)	0.151 (18)	0.044 (8)	−0.025 (12)	0.015 (8)	−0.013 (9)
C10'	0.090 (12)	0.066 (10)	0.046 (7)	0.005 (9)	0.025 (8)	0.016 (7)
N3'	0.092 (3)	0.097 (3)	0.057 (2)	−0.012 (3)	0.020 (2)	−0.009 (2)

### Geometric parameters (Å, °)

**Table:**

Zn1—N5	1.974 (4)	C5—H5	0.9300
Zn1—N4	1.986 (4)	C6—C7	1.499 (6)
Zn1—N2	2.088 (3)	C7—H7A	0.9600
Zn1—N3	2.163 (4)	C7—H7B	0.9600
Zn1—N1	2.195 (3)	C7—H7C	0.9600
S1—C11	1.605 (5)	C8—C9	1.559 (7)
S2—C12	1.601 (5)	C8—H8A	0.9700
N1—C5	1.330 (5)	C8—H8B	0.9700
N1—C1	1.342 (5)	C9—N3	1.388 (6)
N2—C6	1.273 (5)	C9—H9A	0.9700
N2—C8	1.441 (5)	C9—H9B	0.9700
N4—C11	1.154 (5)	C10—N3	1.437 (7)
N5—C12	1.145 (5)	C10—H10A	0.9600
C1—C2	1.383 (5)	C10—H10B	0.9600
C1—C6	1.490 (6)	C10—H10C	0.9600
C2—C3	1.371 (6)	N3—H3A	0.9100
C2—H2	0.9300	C9'—H9'A	0.9700
C3—C4	1.354 (6)	C9'—H9'B	0.9700
C3—H3	0.9300	C10'—H10D	0.9600
C4—C5	1.385 (6)	C10'—H10E	0.9600
C4—H4	0.9300	C10'—H10F	0.9600
			
N5—Zn1—N4	110.56 (16)	C6—C7—H7A	109.5
N5—Zn1—N2	131.51 (14)	C6—C7—H7B	109.5
N4—Zn1—N2	117.70 (15)	H7A—C7—H7B	109.5
N5—Zn1—N3	97.92 (16)	C6—C7—H7C	109.5
N4—Zn1—N3	99.59 (16)	H7A—C7—H7C	109.5
N2—Zn1—N3	79.09 (15)	H7B—C7—H7C	109.5
N5—Zn1—N1	95.51 (13)	N4—C11—S1	179.4 (4)
N4—Zn1—N1	96.97 (14)	N5—C12—S2	178.5 (4)
N2—Zn1—N1	74.85 (12)	N2—C8—C9	107.8 (4)
N3—Zn1—N1	153.44 (15)	N2—C8—H8A	110.2
C5—N1—C1	118.3 (3)	C9—C8—H8A	110.2
C5—N1—Zn1	127.2 (3)	N2—C8—H8B	110.2
C1—N1—Zn1	114.3 (3)	C9—C8—H8B	110.2
C6—N2—C8	124.6 (4)	H8A—C8—H8B	108.5
C6—N2—Zn1	119.6 (3)	N3—C9—C8	111.9 (5)
C8—N2—Zn1	115.5 (3)	N3—C9—H9A	109.2
C11—N4—Zn1	159.8 (4)	C8—C9—H9A	109.2
C12—N5—Zn1	177.7 (4)	N3—C9—H9B	109.2
N1—C1—C2	121.3 (4)	C8—C9—H9B	109.2
N1—C1—C6	114.3 (3)	H9A—C9—H9B	107.9
C2—C1—C6	124.4 (4)	N3—C10—H10A	109.5
C3—C2—C1	119.2 (4)	N3—C10—H10B	109.5
C3—C2—H2	120.4	H10A—C10—H10B	109.5
C1—C2—H2	120.4	N3—C10—H10C	109.5
C4—C3—C2	120.2 (4)	H10A—C10—H10C	109.5
C4—C3—H3	119.9	H10B—C10—H10C	109.5
C2—C3—H3	119.9	C9—N3—C10	119.7 (7)
C3—C4—C5	117.8 (4)	C9—N3—Zn1	109.1 (3)
C3—C4—H4	121.1	C10—N3—Zn1	114.1 (5)
C5—C4—H4	121.1	C9—N3—H3A	104.0
N1—C5—C4	123.3 (4)	C10—N3—H3A	104.0
N1—C5—H5	118.4	Zn1—N3—H3A	104.0
C4—C5—H5	118.4	H9'A—C9'—H9'B	108.1
N2—C6—C1	116.2 (3)	H10D—C10'—H10E	109.5
N2—C6—C7	124.7 (4)	H10D—C10'—H10F	109.5
C1—C6—C7	119.1 (4)	H10E—C10'—H10F	109.5

### Hydrogen-bond geometry (Å, °)

**Table:**

*D*—H···*A*	*D*—H	H···*A*	*D*···*A*	*D*—H···*A*
N3—H3A···S1^i^	0.91	2.66	3.551 (5)	165

Symmetry codes: (i) *x*−1, *y*, *z*.

## References

[bb1] Bruker (2004). *APEX2* and *SAINT* Bruker AXS Inc., Madison, Wisconsin, USA.

[bb2] Li, X.-W. & Qiu, Y. (2008*a*). *Acta Cryst.* E**64**, m113. 10.1107/S1600536807063908 PMC2915066 21200473

[bb3] Li, X.-W. & Qiu, Y. (2008*b*). *Acta Cryst.* E**64**, m218. 10.1107/S1600536807063325 PMC2915145 21200565

[bb4] Panneerselvam, P., Nair, R. R., Vijayalakshmi, G., Subramanian, E. H. & Krishnan, S. (2005). *Eur. J. Med. Chem.* **40**, 225–229. 10.1016/j.ejmech.2004.09.003 15694658

[bb5] Sheldrick, G. M. (2004). *SADABS* University of Göttingen, Germany.

[bb6] Sheldrick, G. M. (2008). *Acta Cryst.* A**64**, 112–122. 10.1107/S0108767307043930 18156677

[bb7] Shi, L., Ge, H.-M., Tan, S.-H., Li, H.-Q., Song, Y.-C., Zhu, H.-L. & Tan, R.-X. (2007). *Eur. J. Med. Chem.* **42**, 558–564. 10.1016/j.ejmech.2006.11.010 17194508

[bb8] Singh, K., Barwa, M. S. & Tyagi, P. (2006). *Eur. J. Med. Chem.* **41**, 147–153. 10.1016/j.ejmech.2005.06.006 16271421

[bb9] Singh, K., Barwa, M. S. & Tyagi, P. (2007). *Eur. J. Med. Chem.* **42**, 394–402. 10.1016/j.ejmech.2006.10.016 17224205

[bb10] Zhong, X., Yi, J., Sun, J., Wei, H.-L., Liu, W.-S. & Yu, K.-B. (2006). *Eur. J. Med. Chem.* **41**, 1090–1092. 10.1016/j.ejmech.2006.05.009 16782235

